# Survival and quality of life of patients with oral and oropharyngeal cancer at 1-year follow-up of tumor resection

**DOI:** 10.1590/S1678-77572010000300015

**Published:** 2010

**Authors:** Maria Gabriela Haye BIAZEVIC, José Leopoldo Ferreira ANTUNES, Janina TOGNI, Fabiana Paula de ANDRADE, Marcos Brasilino de CARVALHO, Victor WÜNSCH-FILHO

**Affiliations:** 1 PhD, Assistant Professor, School of Dentistry, University of São Paulo, São Paulo, SP, Brazil.; 2 PhD, Professor, School of Arts, Science and Humanity, University of São Paulo, São Paulo, SP, Brazil.; 3 Undergraduate student, School of Dentistry, University of São Paulo, São Paulo, SP, Brazil.; 4 MSc, School of Dentistry, University of São Paulo, São Paulo, SP, Brazil.; 5 PhD, Head and Neck Surgery Service, Heliópolis Hospital, São Paulo, SP, Brazil.; 6 PhD, Professor, School of Public Health, University of São Paulo, São Paulo, SP, Brazil.

**Keywords:** Quality of life, Mouth neoplasms, Oropharyngeal neoplasms, Surgery

## Abstract

**Objective:**

This study aimed to assess the survival and life quality evolution of patients subjected to surgical excision of oral and oropharyngeal squamous cell carcinoma.

**Material and Methods:**

Forty-seven patients treated at a Brazilian healthcare unit specialized in head and neck surgery between 2006 and 2007 were enrolled in the study. The gathering of data comprised reviewing hospital files and applying the University of Washington Quality of Life (UW-QOL) questionnaire previously and 1 year after the surgery. Comparative analysis used Poisson regression to assess factors associated with survival and a paired t-test to compare preoperative and 1-year postoperative QOL ratings.

**Results:**

1 year after surgery, 7 patients were not found (dropout of the cohort); 15 had died and 25 fulfilled the UW-QOL again. The risk of death was associated with having regional metastasis previously to surgery (relative risk=2.18; 95% confidence interval=1.09-5.17) and tumor size T3 or T4 (RR=2.30; 95%CI=1.05-5.04). Survivors presented significantly (p<0.05) poorer overall and domain-specific ratings of quality of life. Chewing presented the largest reduction: from 74.0 before surgery to 34.0 one year later. Anxiety was the only domain whose average rating increased (from 36.0 to 70.7).

**Conclusions:**

The prospective assessment of survival and quality of life may contribute to anticipate interventions aimed at reducing the incidence of functional limitations in patients with oral and oropharyngeal cancer.

## INTRODUCTION

“Quality of life” (QOL) is a construct increasingly used to assess health status and the impact of therapeutics in patients with different diseases. In 1994, a panel of researchers from the World Health Organization proposed a unifying and transcultural definition of QOL as “the individual’s perception of his or her position in life, within the cultural context and value system he or she lives in, and in relation to his or her goals, expectations, parameters and social relations”^13^.

QOL is a comprehensive, multidimensional concept, further specified as health-related quality of life (HRQOL) in assessments addressing treatment side effects, physical function and performance. For cancer patients, HRQOL is the subset of QOL that specifically refers to the burden of disease on a patient’s bio-psychosocial profile and how they cope with treatment^7^. HRQOL is currently considered a powerful predictor of mortality and morbidity^14^.

For patients with head and neck (H&N) cancer, the self-oriented HRQOL evaluation is a useful aid to the assessment of therapeutic effectiveness, which otherwise would rely exclusively on endpoint results such as survival and tumor relapse. The assessment of HRQOL allows health professionals to appraise the physical, mental and social impact of therapeutics, and improve their ability to anticipate the patient’s prognosis. Despite advances in diagnosis and treatment, oral and oropharyngeal tumor resection remains associated with disfigurement and dysfunctions that affect essential domains of life. The importance of assessing the self-reported evaluation of functional status and well-being of these patients has been well documented in the literature^6,7,12^.

This study specifically aimed at describing the HRQOL evolution of patients with oral and oropharyngeal cancer 1 year after primary surgery for tumor resection, as a strategy to contribute to the planning of postoperative clinical follow-up.

## MATERIAL AND METHODS

The study sample comprised patients affected by squamous cell carcinoma in the lips, inner aspect (C00.3-C00.9 codes of the International Classification of Diseases, 10^th^ revision), tongue (C01-C02), oral cavity (C03-C06), or oropharynx (C09-C10), which were subjected to primary surgery at the Hospital Heliópolis, between October 2006 and September 2007. This is a large hospital located in the city of São Paulo, Brazil, comprising a referral unit for H&N surgery. Being publicly sponsored, this hospital mostly offers free-of-charge treatment to low-income patients. A dental student (not pertaining to the hospital staff) invited all patients that met study profile to inform their HRQOL status immediately before the primary surgery for tumor resection. The patients completed the University of Washington Quality of Life questionnaire (Portuguese version, UW-QOL, version 4) by themselves, without help of relatives or hospital staff.

This questionnaire has been specifically developed for the QOL assessment of patients with H&N cancer. It comprises general and specific questions addressing relevant HRQOL dimensions for patients with oral and oropharyngeal cancer: pain, appearance, activity, recreation, swallowing, chewing, speech, shoulder function, taste, saliva production, mood, and anxiety. A Likert scale rates answers from 0 to 100, with higher figures indicating improved status^17^. The Portuguese version of this questionnaire (specifically prepared for the Brazilian context) was already validated^15^.

The initial, preoperative information on HRQOL of patients refers to the day of hospitalization for primary surgery for tumor resection. A dynamic search for each patient was performed at the 1-year follow-up of surgery. Patients were classified according to their status: deceased, dropout of the cohort, or available for the evaluation of HRQOL evolution. Scores attributed to the overall status and specific domains of HRQOL were independently assessed before and 1 year after surgery, being subsequently compared by a paired t-test.

This study also used hospital files gathered by the Clinical Genome of Cancer Project^18^ to inform socio-demographic characteristics (gender, age, skin color, and education), behavior (whether patients remained consuming tobacco and alcohol), and their clinical status (tumor localization and TNM classification). Covariates were dichotomously classified for the assessment of survival. Sociodemographic characteristics used categories of gender (females/males), age (<55/>54), skin color (light- and dark-skinned blacks/whites), and education (complete/incomplete basic education, which, in Brazil corresponds to 8 years of formal schooling). Tumor localization differentiated neoplasm affecting anterior (lips, vestibule and floor of mouth, cheek mucosa, hard palate, gum and anterior two-thirds of the tongue) from posterior (base of tongue, soft palate, retromolar area, tonsil and oropharynx) portions of the stomatognathic system. The TNM classification allowed comparing patients with T1/T2 and T3/T4 tumors, and those with and without regional metastasis (N1-2/N0). Current smokers and alcohol consumers were compared with those that never smoked or drank, or interrupted the habit before hospitalization.

Sociodemographic, behavioral and clinical covariates instructed the comparative analysis of survival. This assessment used Poisson regression analysis with robust variance estimation^2^ , which allowed calculating the relative risk of death and their respective 95% confidence intervals. A relative risk higher than the unity suggests that the comparison group had higher risk of death than the reference group. The inverse occurs when the relative risk is lower than the unity; whereas confidence intervals including the unity indicate that survival did not differ between groups.

Statistical analyses used Stata 10 (Stata, Stata Corporation, College Station, Texas, United States of America), 2007. Patients signed a form of informed consent, and ethical approval was given by the Research ethics Committees of the participating institutions (SISNeP N. 0078.0.264.017-05).

## RESULTS

The use of the UW-QOL was well accepted by patients; they were cooperative, and no eligible participant refused to answer. The patients appreciated informing their HRQOL, and they completed the questionnaire without the help of relatives or any proxy respondent. During one full year of monitoring the H&N unit of the hospital, 53 eligible participants were identified for the study; that is, they had oral and oropharyngeal squamous cell carcinoma and were hospitalized for primary surgery. Two patients died during the immediate postoperative period, and four surgeries were cancelled for different reasons. The remaining 47 patients were enrolled in the cohort: 19 patients had tumor in the oral cavity (floor of mouth, gingiva, retromolar area and palate), 12 in the oropharynx, 11 in the tongue and 5 in the inner aspect of the lower lip.

One year after surgery, 7 patients (15%) could not be found and were considered dropout of the cohort. From the remaining patients, 15 (38%) had died and 25 (62%) fulfilled the UW-QOL again. Socio-demographic and behavioral characteristics of patients did not associate with death, although covariates on clinical status did. Patients presenting regional metastasis before surgery (relative risk = 2.18; 95% confidence interval = 1.09-5.17) and those with tumor size T3/T4 (RR=2.30; 95%CI=1.05-5.04) had higher risk of death during the 1-year follow-up of primary surgery (Table 1). A borderline excessive risk of death, at the threshold of statistical significance (p=0.10), was identified for patients with tumors at posterior anatomic sites of the stomatognathic system (RR=1.88; 95%CI=0.89-3.99).

**Table 1:** Risk of death after 1-year follow-up of primary surgery for oral and oropharyngeal cancer (n=40)

**Sociodemographic characteristics**		**Decease**	**Survival**	**Relative risk (95%CI)( 1 )**	**p-value**
					
Gender	female	3	3	1.42	0.464
	male	12	22	(0.56-3.60)	
Age	>54	7	12	0.97	0.936
	<55	8	13	(0.43-2.18)	
Skin color	black	6	7	1.38	0.427
	white	9	18	(0.62-3.09)	
Education	complete basic education	6	5	1.76	0.154
	incomplete basic education	9	20	(0.81-3.81)	
**Behavior**					
Remained smoking		7	12	0.97	0.936
Never smoked/stopped smoking		8	13	(0.43-2.18)	
Remained drinking alcoholic beverages		5	8	1.04	0.931
Never drank/stopped drinking		10	17	(0.44-2.44)	
**Clinical status**					
Tumor localization ( 2 )	posterior sites	7	8	1.88	0.100
	anterior sites	8	17	(0.89-3.99)	
Tumor size ( 3 )	T3/T4	8	7	2.30	0.037
	T1/T2	7	18	(1.05-5.04)	
Regional metastasis	N1/N2	8	5	2.18	0.030
	N0	7	20	(1.09-5.17)	

1 Relative risk and 95% confidence intervals assessed by Poisson regression analysis with robust variance estimation

2 Adjusted by tumor size

3 Adjusted by tumor localization

**Figure 1:**
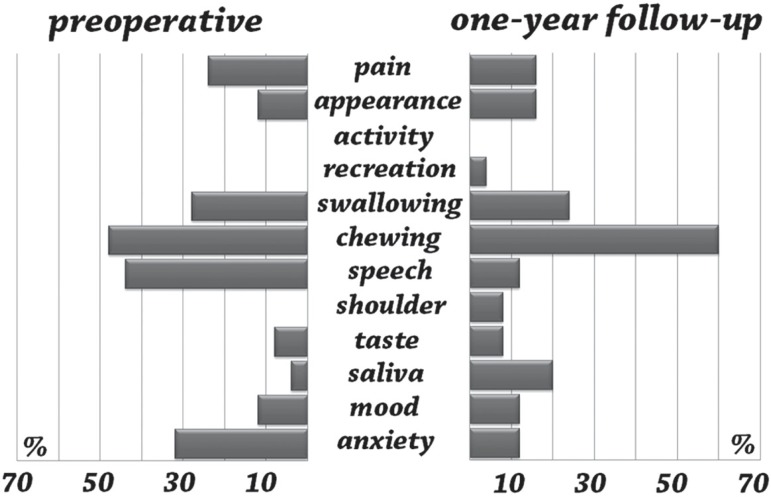
Prevalence of most important complaints for HRQOL-specific domains: preoperative assessment and 1-year follow-up (n=25)

For survivors, chewing (48%), speech (44%), and anxiety (32%) were the most prevalent complaints at the baseline. Chewing (60%), swallowing (24%), and saliva production (20%) were the most relevant complaints at the 1-year follow-up (Figure 1).

Survivors presented a significantly reduced (p=0.006) overall rating of HRQOL at 1-year follow-up, as compared with the preoperative assessment. QOL presented significantly reduced ratings (p<0.05) for activity, recreation, chewing, swallowing, speech, shoulder function and saliva production. Chewing was the QOL domain with largest reduction of rating: from 74.0 preoperatively to 34.0 1 year after surgery. Anxiety was the poorest rating domain before surgery (36.0); however, anxiety was the only domain that increased significantly its average rating, to 70.7, 1 year after surgery (Table 2). The remaining HRQOL domains (pain, appearance, taste and mood) did not present significant reduction of ratings (Table 2).

**Table 2:** Ratings for HRQOL-specific domains: preoperative assessment and 1-year follow-up, longitudinal variation and p-values (paired t-test) (n=25)

**HRQOL Domain**	**Preoperatively**	**1-year follow-up**	**Variation (%)**	**Significance**
				
Pain	76.0	85.0	+ 11.8	p=0.185
Appearance	86.0	77.0	-10.5	p=0.195
Activity	93.0	65.0	-30.1	p=0.002
Recreation	94.0	61.0	-35.1	p=0.003
Swallowing	92.0	64.6	-29.8	p=0.001
Chewing	74.0	34.0	-54.1	p<0.001
Speech	84.1	68.1	-19.1	p=0.003
Shoulder function	97.4	70.4	-27.7	p=0.001
Taste	86.6	82.8	-4.5	p=0.536
Saliva production	89.3	75.0	-16.0	p=0.006
Mood	73.0	68.0	-6.8	p=0.569
Anxiety	36.0	70.7	+96.3	p=0.007
Overall	81.8	68.5	-16.3	p=0.006

Note: Ratings range from 0 (worst subjective function) to 100 (no subjective deficit)

## DISCUSSION

This study identified clinical factors associated with the prognosis of death for patients subjected to primary surgery of oral and oropharyngeal cancer. This study also identified a significant reduction of overall and domain-specific HRQOL ratings at the 1-year follow-up of surgery. These findings are the most relevant results of the study.

Survival analysis usually follows up patients for a longer period and assesses hazard ratios of the time lag between surgery and prospective death. This assessment was prevented by the short period of monitoring, and the reduced number of patients. Survival was exclusively assessed as a categorical outcome to appraise covariates for the risk of dying. Patients with regional metastasis and larger tumors had a higher risk of death during the first year after surgery. Previous studies in the Brazilian context have also reported a lower survival rate for patients presenting poorer clinical profile^4,9^. No participant of the present study presented distant metastasis.

No sociodemographic characteristic was associated with survival. However, the study cannot be considered conclusive in this respect because of its reduced sample size. The comparison of outcomes reported by patients that continued consuming tobacco or alcoholic beverages with those that never smoked or drank, or interrupted the habit when affected by the disease, aimed at assessing the effectiveness of the patient’s support and commitment to the treatment. No differences in survival were observed among patients that remained smoking or drinking after surgery; anyhow, the number of patients currently monitored is too small to allow for inferences on this issue. There is little in the literature regarding the effect of tobacco on postoperative QOL status of patients with oral cancer; however, previous studies that assessed this condition reported absent association^7,12^.

Patients preparing for tumor resection have reasons to be anxious. They are affected by a life-threatening disease, and forthcoming surgery may impact on their quality of life. Indeed, an average 31.1% reduction in the overall HRQOL rating was reported for patients immediately after surgery^3^ ; that is, nearly one third of the patients’ remaining HRQOL, after disease had already subtracted part of their physical and psycho-social functioning. However, anxiety was the only domain that improved its rating in the longitudinal assessment, which suggests that survivors felt relieved and hopeful postoperatively. Most patients renew their state of mind after primary surgery, despite experiencing an immediate deterioration of HRQOL in several physiological domains. Patients with H&N cancer present high levels of depressive symptoms^15^ ; anxiety disorders usually rank highest at diagnosis, mental distress substantially decreases one to three years after surgery^10,11^.

Anxiety was the HRQOL domain ranking the poorest ratings preoperatively. In spite of this, complaints related to chewing and speech were even more prevalent than anxiety during the week that preceded hospitalization for tumor resection. Chewing was the domain ranking the poorest postoperative ratings; a larger reduction of ratings 1 year after surgery affected chewing, activity, recreation and swallowing. A previous study in Brazil stated that, among physiological functions, chewing was the most prevalent complaint of patients with mouth neoplasms^1^. This observation reinforces the importance of dental rehabilitation to patients subjected to surgical resection of oral and oropharyngeal cancer.

There is little or no surprise at all to acknowledge that chewing is largely affected by surgical excision of mouth tissues. However, the large decrement of ratings attributed to this domain is suggestive that this cohort experienced a reduced access to specialized dental rehabilitation after surgery, which highlights the need of integrating the dentist to the multidisciplinary health care team that attends these patients.

Several studies described the postoperative evolution of HRQOL for patients with oral and oropharyngeal tumors and assessed factors associated with improvements in prognosis^5-8,16,19^. These studies reinforce the hypothesis that patients that survived surgery may effectively improve and even recover their HRQOL levels, at least to preoperative ratings. Therefore, the present report of decrease in several specific HRQOL domains after surgery should be taken into careful consideration by medical staff in their effort to anticipate prognosis and design effective treatment protocols.

This study used the UW-QOL questionnaire to describe the postoperative evolution of HRQOL in patients with oral and oropharyngeal cancer. The UW-QOL is a validated, accurate, and internationally accepted survey instrument. Despite this observation, this questionnaire was specifically designed to assess impacts at the H&N region, and may be poorly comprehensive of broader clinical conditions.

The selection of patients exclusively considered only one hospital located in the city of São Paulo, and the sample cannot be considered representative of patients with oral and oropharyngeal cancer in any broader context. As this hospital is a referral health care unit for H&N surgery, some of their patients dwell outside the city of São Paulo, which may have contributed for the relatively large dropout of the cohort: 7 (15%) patients could not be contacted 1 year after surgery. Reduced sample size and the dropout are acknowledged as the main limitations of this study.

The small number of subjects also prevented the assessment of covariates for HRQOL ratings and evolution, which is also acknowledged as a limitation of this study. The outcomes for patients with H&N cancer who survive the initial period after diagnosis and surgery may be more dependent on their comorbidities than on their initial malignant tumor. It was also observe that individuals with poorer socioeconomic status may experience disproportionately higher HRQOL impacts from almost every disease and have poorer prognosis than their better-off counterparts. Having failed to assess factors associated with postoperative HRQOL, this study strongly advocates the conduction of further research involving a larger number of participants, to assess hypotheses of association, which may guide the adoption of preemptive interventions.

## CONCLUSIONS

The prospective assessment of survival and QOL evolution is a useful adjunct for the assessment of prognosis and effectiveness of treatments. Survival was mainly influenced by the clinical status (regional metastasis and tumor size) of patients. Survivors presented significant decrement for the overall and several domain-specific QOL ratings at 1-year follow-up. Chewing was the most relevant complaint of patients. The prospective monitoring of HRQOL may contribute to anticipate interventions aimed to improve survival and reduce the incidence of functional limitations in patients with oral and oropharyngeal cancer.
